# Effects of ultrasound-guided stellate ganglion block on postoperative sore throat and postoperative sleep disturbance after lumbar spine surgery: a randomized controlled trial

**DOI:** 10.1186/s12871-023-02301-y

**Published:** 2023-10-14

**Authors:** Decai Luo, Yanhong Su, Yong Pang

**Affiliations:** Department of Anesthesiology, Affiliated Hospital of North Sichuan Medical College, Nanchong, China

**Keywords:** Stellate ganglion block, Lumbar spine surgery, Postoperative sore throat, Postoperative sleep disturbance

## Abstract

**Background:**

Postoperative sore throat and sleep disturbance are prevalent among patients undergoing spinal surgery, and these conditions can substantially impact their postoperative satisfaction and quality of life. The present study aimed to examine the impact of ultrasound-guided stellate ganglion block (SGB) on the occurrence of postoperative sore throat (POST) and postoperative sleep disturbance (PSD) in patients who undergo lumbar spine surgery under general anesthesia.

**Methods:**

Sixty patients were randomly assigned to either the experimental group (SGB group) or the control group (CG). Both groups received the same induction and maintenance drugs. However, the SGB group received a right SGB under ultrasound guidance 15 min before anesthesia induction, while the CG did not receive any block anesthesia intervention before anesthesia induction. We monitored the incidence and severity of POST at 1, 6, 24, and 48 h after surgery in both groups. Additionally, we evaluated the deep sleep quality score on the first, second, and fifth days after surgery in both groups.

**Results:**

The incidence of POST at 1 h and 6 h after surgery was significantly lower in the SGB group (10.0% and 13.3%) than in the CG (43.3% and 36.7%) (*P* < 0.05). The postoperative sore throat scores of the SGB group (0.10 ± 0.31 and 0.17 ± 0.46) at 1 h and 6 h after surgery were lower than those of the CG (0.57 ± 0.73 and 0.50 ± 0.77) (*P* < 0.05). Moreover, the deep sleep quality score on the first, second, and fifth days after surgery were significantly higher in the CG (5.40 ± 3.37, 4.70 ± 3.19, 4.53 ± 3.44) than in the SGB group (3.87 ± 2.30, 3.13 ± 1.77, 3.03 ± 1.84) (*P* < 0.05).

**Conclusion:**

Ultrasound-guided SGB can reduce the incidence and severity of POST and improve PSD in patients undergoing lumbar spine surgery.

**Trial registration:**

This study was registered on Chinese Clinical Trial Registry, (ChiCTR2200065279) on 01/11/2022.

## Introduction

Stellate ganglion block (SGB) is a reversible technique that involves blocking the sympathetic nerves in the neck and their distribution area. Clinical research has shown that SGB is effective in pain suppression, regulation of autonomic nervous system dysfunction, and reduction of the adverse stress response caused by pituitary-adrenal gland hormonal secretion. It is used to alleviate postoperative pain, address arrhythmias, manage psychiatric disorders, and treat vasospastic syndromes [1–5]. Consequently, SGB is considered to have a high level of safety and is extensively employed during the perioperative period for surgical patients [4, 6].

During lumbar spine surgery, patients must maintain a prone position for an extended period. Moreover, the transition from a supine to a prone position may displace the endotracheal tube in relation to the airway, which can increase the incidence of postoperative sore throat (POST) by more than 60% [7]. This displacement may also result in increased hemodynamic fluctuations [8].

Postoperative sleep disturbance (PSD) can occur after surgery for various reasons, such as pain from the surgical incision site on the back and dysfunction of the autonomic nervous system. The incidence of PSD can be as high as 40% to 60% [9, 10].

Prior studies have primarily investigated the effectiveness of SGB during lumbar surgery, whereas this study aimed to examine whether SGB can effectively alleviate postoperative throat pain and improve postoperative sleep disorders in patients undergoing lumbar surgery. SGB may represent an effective approach to reducing adverse reactions and enhancing patient comfort during the recovery period.

## Materials and methods

### General information

This study was a prospective, randomized controlled trial that received approval from the Ethics Committee of the Affiliated Hospital of North Sichuan Medical College (Approval No: 2022ER403-1) on 14/10/2022 and was registered in the Chinese Clinical Trial Registry (ChiCTR2200065279) on 01/11/2022. Prior to the experiment, patients and their families signed an informed consent form. A total of 60 patients with lumbar spondylosis who underwent lumbar surgery under general anesthesia at the Affiliated Hospital of North Sichuan Medical College between October 2022 and January 2023 were enrolled in the study. The patients were randomly allocated into either the experimental group (30 patients) or the control group (30 patients). There were no significant differences in the demographic profile between the two groups (*P* > 0.05), indicating comparability.

The inclusion criteria were as follows: (1) patients who underwent lumbar surgery with a clear preoperative diagnosis; (2) patients aged between 45 and 65 years old; (3) patients classified as ASA grade I∼II; and (4) patients who did not undergo a preoperative laryngoscopy examination.

The exclusion criteria were as follows: (1) preoperative throat pain or hoarseness. (2) a history of upper respiratory tract infection within 2 weeks; (3) anticipated difficulty in tracheal intubation (Mallapati score of III or IV, potential facial deformities, or mouth opening < 3 fingers); (4) severe functional disorders of the heart, liver, kidney, or other organs before surgery; (5) allergy to local anesthetics used in the study, puncture site infection, or abnormal coagulation function; and (6) cognitive dysfunction, hearing impairment, language barriers, history of mental illness, difficult communication or cooperation due to psychiatric disorders.

The withdrawal criteria were as follows: (1) failed SGB prior to the surgery and (2) unable to achieve successful endotracheal intubation on the first attempt.

### Treatment regimen

Both groups of patients underwent intravenous inhalation compound general anesthesia for surgery, and the administration of medication and endotracheal intubation were both performed by the same anesthesiologist. Upon entering the operating room, routine tests were conducted, including heart rate (HR), mean arterial pressure (MAP), pulse oxygen saturation (SpO2), and electrocardiogram (ECG). A peripheral intravenous infusion channel was established. Anesthesia induction was performed in sequence with sufentanil 0.3 μg/kg, propofol 1.5–2.0 mg/kg, and atracurium besylate 0.15 mg/kg. After 3–5 min of atracurium besylate injection, muscle relaxation was achieved, and a Flexometallic tube with a size of I.D6.5 was used for female patients, and a Flexometallic tube with a size of I.D7.0 was used for male patients. The patient was then turned from a supine position to a prone position, with the head remaining relatively still in comparison to the body during the turning process. Once the turning process was complete, the head was placed on a silicone pillow.

A three-way tube was utilized to connect the cuff of the tracheal tube, a 10 ml syringe, and a pressure gauge for monitoring and maintaining cuff pressure at 20–30 cmH_2_O. The cuff was checked for leakage every 30 min, the cuff pressure remained within the acceptable range. Following intubation, mechanical ventilation was initiated, and ventilator parameters were adjusted to achieve a tidal volume of 6–8 ml/kg, respiratory rate of 12–16 breaths/min, and end-tidal CO_2_ controlled at 35–45 mmHg. During the surgery, anesthesia was sustained using 2.0%-3.0% sevoflurane, and atracurium besylate and sufentanil were intermittently administered as required. Vasopressor medications were employed to manage fluctuations in blood pressure.

The administration of atracurium besylate injection was ceased 40 min prior to the conclusion of the surgery, and a final dose of 10 μg sufentanil was administered 30 min before the end of the surgery. Following completion of the turning process, sevoflurane was discontinued, and the patient was given pure oxygen for 10–15 min to aid in lung cleaning. Suction care was performed and extubation criteria were confirmed before removing the tracheal tube. In the postanesthesia care unit (PACU), routine monitoring was performed, and the patient was transferred to the ward after satisfying the discharge criteria.

The SGB group received an ultrasound-guided stellate ganglion block 15 min prior to anesthesia induction. An ultrasound machine was utilized to identify the position of the longus colli muscle at the C6 cervical level. Numerous studies have demonstrated that a right stellate ganglion block more effectively suppresses sympathetic nerve excitability, reduces the stress response, and alleviates fluctuations in heart rate and blood pressure. These outcomes aim to stabilize intraoperative hemodynamics [11]. Following the application of local anesthesia to the right puncture site, an in-plane needle approach was implemented to puncture the muscle surface. Upon confirming the absence of cerebrospinal fluid, blood, or gas upon aspiration, 5 mL of 0.5% bupivacaine was injected to perform an SGB. The occurrence of Horner's syndrome on the blocked side served as confirmation of a successful SGB. After successful block implementation, the patient's vital signs were closely monitored for any complications.

The control group underwent stellate ganglion localization under ultrasound guidance prior to anesthesia induction, but no invasive blocking anesthesia intervention was carried out.

### Analgesic regimen

Both patient groups were provided intravenous patient-controlled analgesia (PCA) for postoperative pain management. The analgesic regimen was comprised 150 µg sufentanil, 200 mg flurbiprofen ester, and 5 mg tropisetron, which were diluted to 150 mL with normal saline. The PCA parameters were established as follows: a background infusion rate of 1.5 mL/h, a single dose of 0.5 mL, a lockout time of 15 min, and a total duration of 24 h. No other analgesic medications were administered as interventions after surgery.

### Observation indicators

(1) The incidence and severity of postoperative sore throat (POST) were embodied in the postoperative sore throat score, a four-level rating scale used to assess the severity of postoperative throat pain [12] (0 points: no throat pain at any time after the surgery; 1 point: mild pain; 2 points: moderate throat pain; 3 points: severe throat pain causing changes in voice or hoarseness.). Postextubation cough (PEC), hoarseness of voice (HOV), and postoperative nausea and vomiting (PONV) were also recorded for both groups of patients at 1 h, 6 h, 24 h, and 48 h after surgery. (2) The quality of sleep was assessed using the "Sleep Quality Assessment Scale" on the day before surgery, and one, two, and five days after surgery for both groups of patients. The Chinese Sleep Research Society formulated the "Sleep Quality Assessment Scale" based on the World Health Organization (WHO). A total score of less than 4 indicates good sleep quality, a score between 4–6 indicates poor sleep quality, and a score higher than 6 indicates very poor sleep quality, which can negatively impact physical and mental health. The higher the score is, the worse the sleep quality [13]. (3) The duration of the endotracheal tube (insertion to removal), anesthesia time (induction, maintenance, recovery), prone position time, and surgical time were recorded for both groups of patients. (4) The changes in heart rate (HR), systolic blood pressure (SBP), diastolic blood pressure (DBP), and mean arterial pressure (MAP) were collected in the two groups of patients at the following time points: before induction (T0), before intubation (T1), immediately after intubation (T2), immediately after turning from the supine to prone position (T3), at the start of skin incision (T4), immediately after turning from the prone to supine position (T5), immediately after extubation (T6), and 5 min after extubation (T7). (5) The levels of C-reactive protein (CRP) and interleukin-6 (IL-6) were measured in both groups of patients one day before and one day after surgery.

### Sample size calculation

Assuming that a 20% decrease in POST incidence is clinically significant, experimental results from 20 lumbar spine surgery patients were used to estimate the sample size using PASS software. A type I error rate of α = 0.05 and a test power of β = 90% were set, and at least 26 samples were needed in each group. Taking into account potential loss to follow-up during the study (10%), 30 patients were recruited per group.

### Data analysis

Data analysis was performed using SPSS 26.0 statistical software. Continuous data are expressed as the mean ± standard deviation (x ± s), and intergroup comparisons were performed using t tests. Within-group comparisons at different time points were performed using repeated measures analysis of variance. Categorical data are expressed as proportions (%), and intergroup comparisons were performed using chi-square tests and exact probability methods. A *P* value < 0.05 was considered statistically significant.

## Results

### Demographic profile

**Fig. 1:**
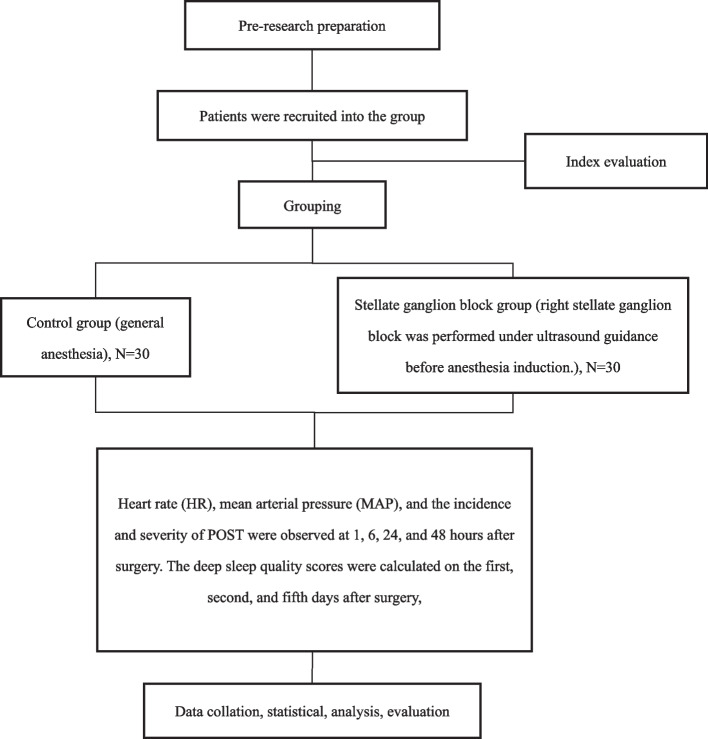
Technology roadmap

The experimental study included a total of 60 patients, none of whom withdrew from the study (the study flow diagram is displayed in Fig. 1). The demographic profile of the patients, including weight, height, age, sex, and ASA classification, exhibited no significant differences between the two groups. The differences in anesthesia induction time, anesthesia maintenance time, anesthesia recovery time, endotracheal intubation time, surgical time, and prone position time between the two groups were not statistically significant (*P* > 0.05), as illustrated in Table 1.

**Table 1:** Demographic profile of two groups

Project	SGB Group(*N* = 30)	CG(*N* = 30)	*P*
Weight/kg	64.07 ± 9.73	68.20 ± 10.33	0.116
Height/cm	162.86 ± 7.38	162.83 ± 8.00	0.987
Age/years	52.87 ± 9.69	51.47 ± 8.89	0.562
Male/female	19/11	18/12	0.791
ASAI/II	15/15	11/19	0.297
Anesthesia induction time/min	6.43 ± 1.43	5.87 ± 1.45	0.134
Anesthesia maintenance time/min	137.07 ± 48.33	143.57 ± 46.50	0.598
Anesthesia recovery time/min	18.87 ± 4.04	20.00 ± 5.22	0.351
Endotracheal intubation time/min	155.93 ± 49.01	163.60 ± 48.98	0.547
Surgical time/min	120.43 ± 48.16	129.87 ± 46.64	0.444
Prone position time/min	141.83 ± 49.07	149.57 ± 48.61	0.542

The SGB group refers to the stellate ganglion block group; The CG refers to the control group; The symbol ∗ represents the comparison between the SGB group and the CG, with *P* < 0.05

### Comparison of postoperative sore throat

The incidence of POST in the SGB group was significantly lower than that in the CG at 1 h and 6 h after general anesthesia (10.0% and 13.3%, respectively, compared to 43.3% and 36.7%, respectively). The mean postoperative sore throat scores of the SGB group (0.10 ± 0.31 and 0.17 ± 0.46, respectively) were lower than those of the CG (0.57 ± 0.73 and 0.50 ± 0.77, respectively), and the differences were statistically significant effect (P < 0.05). However, at 24 h and 48 h after general anesthesia, there was no statistically significant difference in the incidence or postoperative sore throat score of POST between the SGB group and the CG (P > 0.05), as presented in Tables 2 and 3

**Table 2:** Comparison of incidence of postoperative sore throat among two groups [n(%)]

Project	SGB Group(*N* = 30)	CG(*N* = 30)	*P*
1 h after surgery	3(10.0)	13(43.3)	0.004^∗^
6 h after surgery	4(13.3)	11(36.7)	0.037^∗^
24 h after surgery	2(6.7)	6(20.0)	0.255
48 h after surgery	2(6.7)	4(13.3)	0.667

The SGB group refers to the stellate ganglion block group; The CG refers to the control group; The symbol ∗ represents the comparison between the SGB group and the CG, with P < 0.05

**Table 3:** Comparison of the postoperative sore throat scores among two groups

Project	SGB Group(*N* = 30)	CG(*N* = 30)	*P*
1 h after surgery	0.10 ± 0.31	0.57 ± 0.73	0.002^∗^
6 h after surgery	0.17 ± 0.46	0.50 ± 0.77	0.048^∗^
24 h after surgery	0.07 ± 0.25	0.23 ± 0.50	0.111
48 h after surgery	0.10 ± 0.40	0.13 ± 0.35	0.732

The SGB group refers to the stellate ganglion block group; The CG refers to the control group; The symbol ∗ represents the comparison between the SGB group and the CG, with *P* < 0.05

### Comparison of postoperative throat complications

Table 4 shows that the incidence of postoperative hoarseness in the SGB group was significantly lower than that in the CG at 1 h, 6 h, 24 h, and 48 h after surgery (46.7%, 50.0%, 36.7%, and 23.3% vs. 83.3%, 80.0%, 73.3%, and 63.3%, respectively; *P* < 0.05). In terms of postoperative cough, there was no significant difference between the two groups at 1 h, 6 h, and 48 h after surgery. However, the incidence of postoperative cough at 24 h after surgery in the SGB group was significantly lower than that in the CG (13.3% vs. 36.7%, *P* < 0.05), as shown in Table 5. Additionally, there was no significant difference in the incidence of POVN between the SGB group and the CG at any time point after surgery (6.7%, 3.3%, 3.3%, and 3.3% vs. 10.0%, 3.3%, 6.7%, and 6.7%, respectively), as shown in Table 6.

**Table 4:** Comparison of incidence of postoperative hoarseness among two groups [n(%)]

Project	SGB Group(*N* = 30)	CG(*N* = 30)	*P*
1 h after surgery	14(46.7)	25(83.3)	0.003^∗^
6 h after surgery	15(50.0)	24(80.0)	0.015^∗^
24 h after surgery	11(36.7)	22(73.3)	0.004^∗^
48 h after surgery	7(23.3)	19(63.3)	0.002^∗^

The SGB group refers to the stellate ganglion block group; The CG refers to the control group; The symbol ∗ represents the comparison between the SGB group and the CG, with *P* < 0.05

**Table 5:** Comparison of incidence of postoperative cough among two groups [n(%)]

Project	SGB Group(*N* = 30)	CG(*N* = 30)	*P*
1 h after surgery	4(13.3)	1(3.3)	0.350
6 h after surgery	4(13.3)	10(33.3)	0.067
24 h after surgery	4(13.3)	11(36.7)	0.037^∗^
48 h after surgery	6(20.0)	7(23.3)	0.754

The SGB group refers to the stellate ganglion block group; The CG refers to the control group; The symbol ∗ represents the comparison between the SGB group and the CG, with *P* < 0.05

**Table 6:** Comparison of incidence of postoperative nausea and vomiting among two groups [n(%)]

Project	SGB Group(*N* = 30)	CG(*N* = 30)	*P*
1 h after surgery	2(6.7)	3(10.0)	1.000
6 h after surgery	1(3.3)	1(3.3)	1.000
24 h after surgery	1(3.3)	2(6.7)	1.000
48 h after surgery	1(3.3)	2(6.7)	1.000

The SGB group refers to the stellate ganglion block group; The CG refers to the control group; The symbol ∗ represents the comparison between the SGB group and the CG, with *P* < 0.05

### Comparison of deep sleep quality scores

Table 7 shows that there was no significant difference in the deep sleep quality score between the SGB group and the CG before surgery (*P* > 0.05). However, on the 1st, 2nd, and 5th days after surgery, the deep sleep quality score in the SGB group was significantly lower than that in the CG (*P* < 0.05).

**Table 7:** Comparison of incidence of deep sleep quality score among two groups

Project	SGB Group(*N* = 30)	CG(*N* = 30)	*P*
Before surgery	2.97 ± 1.99	3.70 ± 2.43	0.207
On the 1st day after surgery	3.87 ± 2.30	5.40 ± 3.37	0.045^∗^
On the 2nd day after surgery	3.13 ± 1.77	4.70 ± 3.19	0.023^∗^
On the 5th day after surgery	3.03 ± 1.84	4.53 ± 3.44	0.041^∗^

The SGB group refers to the stellate ganglion block group; The CG refers to the control group; The symbol ∗ represents the comparison between the SGB group and the CG, with *P* < 0.05

### Comparison of intraoperative hemodynamics

There was no significant difference in MAP or HR between the SGB group and the CG at T0, T1, or T7 (*P* > 0.05). However, at T2 and T3, the MAP and HR in the SGB group were significantly lower than those in the CG (*P* < 0.05). On the other hand, at T4, T5, and T6, the HR in the SGB group was significantly lower than that in the CG (*P* < 0.05), and there was no significant difference in MAP between the two groups (*P* > 0.05), as shown in Table 8.

**Table 8:** Comparison of hemodynamics among two groups

Project	SGB Group(*N* = 30)	CG(*N* = 30)	*P*
T0(MAP)	102.26 ± 10.63	103.47 ± 12.20	0.683
T0(HR)	79.00 ± 11.79	80.23 ± 14.96	0.724
T1(MAP)	80.81 ± 11.78	82.44 ± 17.25	0.670
T1(HR)	73.30 ± 11.30	77.03 ± 12.03	0.221
T2(MAP)	102.00 ± 17.83	114.88 ± 25.37	0.027^∗^
T2(HR)	88.90 ± 17.18	100.27 ± 13.21	0.006^∗^
T3(MAP)	89.90 ± 11.58	101.16 ± 18.17	0.006^∗^
T3(HR)	77.97 ± 13.05	88.87 ± 13.91	0.003^∗^
T4(MAP)	94.84 ± 13.09	100.06 ± 12.30	0.118
T4(HR)	72.13 ± 11.46	78.83 ± 13.22	0.040^∗^
T5(MAP)	102.40 ± 12.70	106.62 ± 17.69	0.293
T5(HR)	81.87 ± 12.59	89.87 ± 15.47	0.032^∗^
T6(MAP)	102.83 ± 10.52	102.44 ± 19.82	0.925
T6(HR)	88.37 ± 11.47	96.13 ± 13.40	0.019^∗^
T7(MAP)	97.84 ± 11.27	96.60 ± 16.07	0.730
T7(HR)	81.97 ± 9.33	83.07 ± 12.98	0.708

The SGB group refers to the stellate ganglion block group; The CG refers to the control group; The symbol ∗ represents the comparison between the SGB group and the CG, with *P* < 0.05

### Comparison of other data

Table 9 shows that there was no significant difference in the intraoperative use of sufentanil, propofol, or cisatracurium besylate; blood loss; SAS score at extubation; IL-6 and CRP levels before and after surgery; or VAS score at 1 h, 6 h, and 24 h after surgery between the SGB group and the CG (*P* > 0.05). However, the VAS score at 48 h after surgery in the SGB group was significantly lower than that in the CG (*P* < 0.05).

**Table 9:** Comparison of other data among two groups

Project	SGB Group(*N* = 30)	CG(*N* = 30)	*P*
Dose of sufentanil/μg	43.83 ± 7.62	45.17 ± 6.08	0.457
Dose of propofol/mg	133.66 ± 27.85	144.66 ± 24.03	0.107
Dose of cisatracurium besylate/mg	11.53 ± 3.73	12.37 ± 3.10	0.351
Amount of blood loss/ml	295.00 ± 156.11	376.67 ± 337.26	0.234
IL-6 level before surgery	5.43 ± 5.83	12.34 ± 21.07	0.238
CRP level before surgery	6.62 ± 9.55	7.40 ± 13.80	0.814
IL-6 level after surgery	26.41 ± 25.98	20.85 ± 21.45	0.392
CRP level after surgery	17.75 ± 14.35	29.30 ± 42.40	0.169
VAS score 1 h after surgery	2.67 ± 0.99	3.13 ± 1.16	0.101
VAS score 6 h after surgery	2.70 ± 0.79	3.03 ± 0.96	0.149
VAS score 24 h after surgery	2.27 ± 0.78	2.60 ± 0.72	0.093
VAS score 48 h after surgery	1.77 ± 0.81	2.33 ± 0.84	0.011^∗^
SAS score at extubation	3.87 ± 0.34	4.17 ± 0.91	0.101

The SGB group refers to the stellate ganglion block group; The CG refers to the control group; The symbol ∗ represents the comparison between the SGB group and the CG, with *P* < 0.05

## Discussion

Lumbar spine diseases are prevalent among middle-aged and elderly individuals in China. The primary symptoms include intermittent claudication and pain in the legs and lower back. In severe cases, these symptoms can significantly impact daily life. In clinical practice, when conservative treatments prove ineffective, surgical interventions are typically performed [14]. The posterior approach is commonly preferred by surgeons for lumbar spine surgery, requiring patients to maintain a prone position throughout the operation.

During anesthesia induction, the patient needed to be in a supine position for intubation and then rotated from the supine to the prone position while still intubated. Studies have shown that more than 90% of patients experience tracheal tube displacement during the rotation process, with nearly half of them having a displacement of more than 10 mm. In addition, more than 80% of patients experience changes in tracheal cuff pressure [15, 16]. Displacement of the tracheal tube and fluctuations in tracheal cuff pressure are significant contributors to the development of postoperative sore throat, hoarseness, and cough [17]. As a result, surgeries performed in this position have a higher incidence and severity of postoperative airway complications compared to surgeries performed in the general supine position.

POST is a frequent complication after surgery that is directly linked to tracheal intubation. The pressure and friction of the laryngoscope and tracheal tube during intubation can harm the mucosal tissue of the pharynx and larynx, triggering an inflammatory response that leads to the development of POST [18]. POST has an incidence rate ranging from 30 to 70%, which frequently results in a decrease in patients' postoperative quality of life and satisfaction with their recovery [19]. Although there are multiple drugs and methods available to treat POST, the treatment's effectiveness is frequently unsatisfactory. Consequently, reducing the incidence of POST is of the utmost importance [20].

Research has demonstrated that postoperative sleep disorders in orthopedic surgery patients are primarily attributable to physiological and psychological factors. These factors include postoperative incisional pain, restricted physical activity, heightened mental stress, apprehension regarding inadequate postoperative recovery, and discomfort with the hospital environment [21]. Patients who undergo lumbar spine surgery must remain in a supine position for an extended period after the procedure. The surgical incision in the lumbar back area bears the pressure of the patient's entire body weight, resulting in significant pain. This pain is one of the reasons for the high incidence of postoperative sleep disorders in lumbar spine surgery patients [10].

SGB, recognized by most clinical doctors for its safety and efficacy as a sympathetic nerve block, has been extensively utilized [2]. As ultrasound imaging technology has advanced and matured, ultrasound-guided SGB has demonstrated higher safety and success rates than traditional blind puncture. This approach has resulted in a reduced incidence of adverse reactions and complications. Consequently, in this experiment, stellate ganglion block was performed under ultrasound guidance [2, 22].

The study results showed that both groups experienced the most significant POST within 6 h after surgery, and the incidence of POST in the SGB group was significantly lower than that in the CG at 1 h and 6 h after surgery. These findings demonstrate that SGB can effectively alleviate the discomfort and pain associated with throat intubation and enhance postoperative patient comfort. Furthermore, preoperative SGB can effectively reduce the incidence of postoperative hoarseness, but its impact on postoperative cough and PONV is minimal. Although the incidence of POST in the SGB group was slightly lower than that in the CG at 24 h and 48 h after surgery, the results were not statistically significant. This may be due to the use of corticosteroids by surgeons during surgery, which resulted in a lower incidence of POST in the CG than in previous reports [23, 24].

The mechanism by which SGB reduces POST may be related to the extensive distribution of stellate ganglion fibers and the blocking of excitatory conduction of the posterior sympathetic nerves in the throat and tracheal mucosa. This effect prevents sympathetic function from effectively acting on corresponding organs and tissues. Additionally, stellate ganglion block can modify the process of inflammation triggered by tissue injury, inhibit the migration of white blood cells to the inflammation site, and reduce the release of inflammatory factors. This approach can also decrease the body's inflammatory response and oxidative stress damage, thereby preserving tissue cell integrity.

We collected and compared data on the levels of C-reactive protein (CRP) and interleukin-6 (IL-6) in the two groups, but found no statistically significant difference between them. These findings are inconsistent with some previous clinical trials [25, 26]. This may be attributed to the fact that the study did not limit the surgical method used in among the patients. Surgical interventions for the lumbar spine can involve single procedures, such as laminectomy, discectomy, or pedicle screw fixation, or a combination of multiple procedures. The number and location of spinal segments requiring surgery may differ among patients, leading to varying degrees of surgical trauma. Consequently, the severity of postoperative inflammatory reactions and the amount of inflammatory factors released may also vary among patients.

Optimal postoperative sleep quality can expedite patient recovery, decrease hospitalization duration, and alleviate the economic burden on patients [27, 28]. Improving postoperative sleep quality is crucial in lumbar spine surgeries where postoperative sleep disorders are common. SGB can repair and reconstruct the autonomic nervous system and neuroendocrine immune system [29]. Clinical studies have demonstrated that preoperative administration of SGB effectively alleviates postoperative sleep disorders [30, 31]. Our study revealed that patients who received preoperative SGB had significantly lower scores for deep sleep quality on the first, second, and fifth days after surgery compared to the control group. Surgical trauma is not the sole factor that contributes to postoperative sleep disorders. Severe anxiety and depression, immune reaction disorders, and disturbances in circadian rhythm can also cause difficulty in falling asleep for patients [32]. Preoperative SGB can decrease sympathetic nervous system activity, alleviate sleep disorders, and enhance postoperative recovery in orthopedic surgery patients.

We did not find conclusive evidence in this study to support the hypothesis that SGB effectively alleviates perioperative pain. This finding contradicts traditional beliefs and may be because the observed postoperative incision pain was primarily caused by surgical trauma, rather than sympathetic nervous system dysfunction. However, the SGB group did exhibit a decrease in VAS scores at 48 h after surgery, possibly attributable to the anti-inflammatory effect of SGB during the wound recovery phase. One limitation of our study is that we did not measure inflammatory factors in patients again at the 48-h mark after surgery.

Our study also has other limitations. The patients in this study underwent various types of surgeries and had a wide age range, but the sample size was relatively small. In future studies, we plan to include larger sample sizes and to stratify patients based on surgery type and age to better understand the effects of SGB at different levels. Certain factors were not recorded or compared in this study, such as the degree of tracheal tube displacement before and after patient position changes during surgery, and the use of fiber bronchoscopy to identify any injury or irritation to the pharynx during surgery. Additionally, the PCA configuration did not employ individualized schemes based on individual differences, which may have impacted some postoperative experimental results.

## Conclusion

Ultrasound-guided SGB is an effective intervention to reduce the incidence and severity of postoperative sore throat in lumbar surgery patients. It also alleviates postoperative sleep disturbances by inhibiting the excitation of the autonomic nervous system. Therefore, ultrasound-guided SGB can improve patient comfort and enhance the quality of postoperative recovery.

## Data Availability

The datasets generated and/or analyzed during this study are not publicly available due to institutional policy on data confidentiality but are available from the corresponding author on reasonable request.

## Abbreviations

SGB: Stellate ganglion block
POST: Postoperative sore throat
PSD: Postoperative sleep disturbance
PEC: Postextubation cough
PONV: Postoperative nausea and vomiting
HOV: Hoarseness of voice
ASA: American Society of Anesthesiologists
WHO: World Health Organization
HR: Heart rate
MAP: Mean arterial pressure
SpO_2_: Pulse oxygen saturation
ECG: Electrocardiogram
PACU: Postanesthetic care unit
SBP: Systolic blood pressure
DBP: Diastolic blood pressure
PCA: Patient-controlled analgesia
VAS: Visual analog scale
SAS: Sedation-Agitation Scale
